# Derivation of oocyte-like cells from putative embryonic stem cells and parthenogenetically activated into blastocysts in goat

**DOI:** 10.1038/s41598-020-66609-2

**Published:** 2020-06-22

**Authors:** Hruda Nanda Malik, Dinesh Kumar Singhal, Sikander Saini, Dhruba Malakar

**Affiliations:** Animal Biotechnology Centre, National Dairy Research Institute, Karnal, 32001 India

**Keywords:** Embryonic germ cells, Embryonic stem cells

## Abstract

Germ cells are responsible for the propagation of live animals from generation to generation, but to surprise, a steep increase in infertile problems among livestock poses great threat for economic development of human race. An alternative and robust approach is essential to combat these ailments. Here, we demonstrate that goat putative embryonic stem cells (ESCs) were successfully *in vitro* differentiated into primordial germ cells and oocyte-like cells using bone morphogenetic protein-4 (BMP-4) and trans-retinoic acid (RA). Oocyte-like cells having distinct zonapellucida recruited adjacent somatic cells in differentiating culture to form cumulus-oocyte complexes (COCs). The putative COCs were found to express the zonapellucida specific (ZP1 and ZP2) and oocyte-specific markers. Primordial germ cell-specific markers VASA, DAZL, STELLA, and PUM1 were detected at protein and mRNA level. In addition to that, the surface architecture of these putative COCs was thoroughly visualized by the scanning electron microscope. The putative COCs were further parthenogenetically activated to develop into healthy morula, blastocysts and hatched blastocyst stage like embryos. Our findings may contribute to the fundamental understanding of mammalian germ cell biology and may provide clinical insights regarding infertility ailments.

## Introduction

Germ cells hold an idiosyncratic and crucial position in the biorhythms of animals as they pass the genome onto the succeeding generation^1^. In mammals the primordial germ cells (PGCs) are derived from proximal epiblast of a developing embryo^2^. Once developed, PGCs drifted through dorsal mesentery of hindgut and invade the genital ridge of developing fetus in mouse^3^. Large size, low nucleo-cytoplasm ratio, distinct nuclear borders, and granular nuclear chromatin are the important characters of PGCs^4^.

Embryonic stem cells (ESCs) are emanated from blastocyst stage embryos in mammals with unlimited proliferative capacity and multi lineages differentiation ability under suitable culture onditions^5–8^. These cells hold a wide prospective to bestead not only as *in vitro* model system to extrapolate the mechanism of gametogenesis but also a cell based method of inducing mature gametes. Derivation of mature germ cells from ESCs was obtained in two ways: first, spontaneous differentiation of adherent cultures after discarding feeder layers and leukemia inhibitory factor^9–11^ and second, embryoid body-mediated germ cell production^12–17^. Moreover several aspects in germ cells genesis from ESCs i.e. lack of reproducibility and validity of ESCs-derived germ cell identity were not well established^18–20^.

The definitive markers of female germ cells in both XX and XY cell lines is expressed during ESCs development^12,13^. The prevailing hypothesis that regardless of the sex of germ cells, they are inherently destined to undergo meiosis and develop as oocytes, unless otherwise prevented by factors inhabiting meiosis from doing so^1^. Although it has been shown to be possible to distinguish cells resembling germ cells from *in-vitro* ESCs in mice^9,13,21^ humans^18,20^ and primates^16^ to our knowledge, the current investigation is the first to classify ESCs-derived germ cells and investigate their potential for growth in goats. The derivation of germ cells or oocytes from ESCs is a model for studying the molecular basis of the development of germ cells and may one day promote the technology of somatic cell nuclear transfer and infertility treatments.

## Results

### Sexing of putative ESCs

Goat ESCs formed dense and compact colonies after 5 days of culture (Supplementary Fig. 1A). In addition to that, the clumps of ESCs colonies in hanging drop culture developed into three-dimensional mass known as embryoid bodies (Supplementary Fig. 1B). To investigate the genotype of ESCs colonies, the isolated DNA was subjected to RT-PCR amplification using Amelogenin and SRY genes. DNA of Y-chromosome of ESCs yielded a 162 bp product of the SRY gene and X-chromosome resulted in a negative amplification (Supplementary Fig. 1C).

Nevertheless, in order to avoid the incorrect SRY amplification, the DNA of the ESCs produced two amplified products for the amelogenin gene, such as Y- chromosome (202 bp) and X-chromosome (262 bp), thus confirming the genotype of the ESCs as XY- or male (Supplementary Fig. 1C). As a positive control, male goat fibroblast cells developed a 162 bp product for the SRY gene while female fibroblast cells did not produce an amplified product (Supplementary Fig. 1C). Similarly, male fibroblast cells produced two different AMELX (262 bp) and AMELY (202 bp) amplified products, but only one AMELX gene product (262 bp) was produced by female fibroblast cells.

### Derivation of oocytes and COCs like cells from putative ESCs

**Figure 1:**
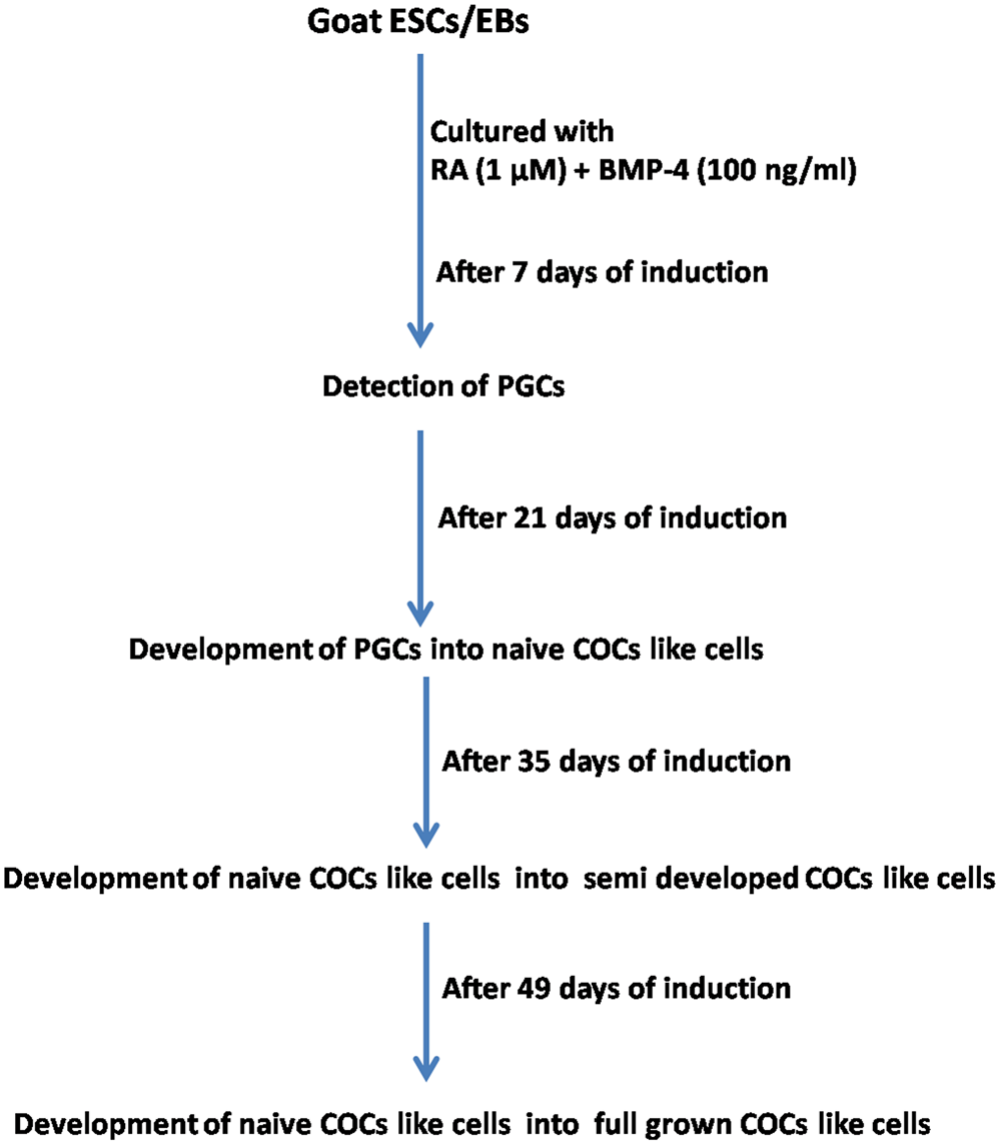
Schematic flow chart illustrating the culture and differentiation protocol for germ cell differentiation from goat putative ESCs.

**Figure 2:**
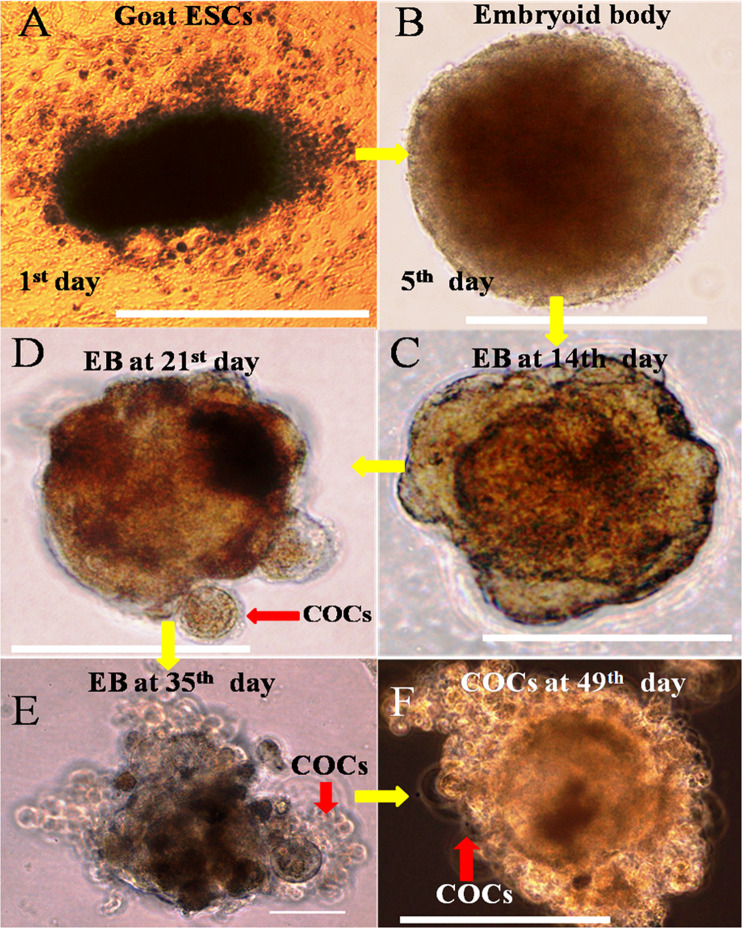
*In vitro* differentiation goat putative ESCs derived EB into COCs like cells. **(A)** ESCs at 1^st^ day of differentiation. **(B)** ESCs-derived EB at 5^th^ day of differentiation. **(C)** EB at 14^th^ day. **(D)** EB at 21^st^ day **(E)** EB at 35^th^ day. **(F)** COCs at 49^th^ day. Scale bar 100 µm.

**Figure 3:**
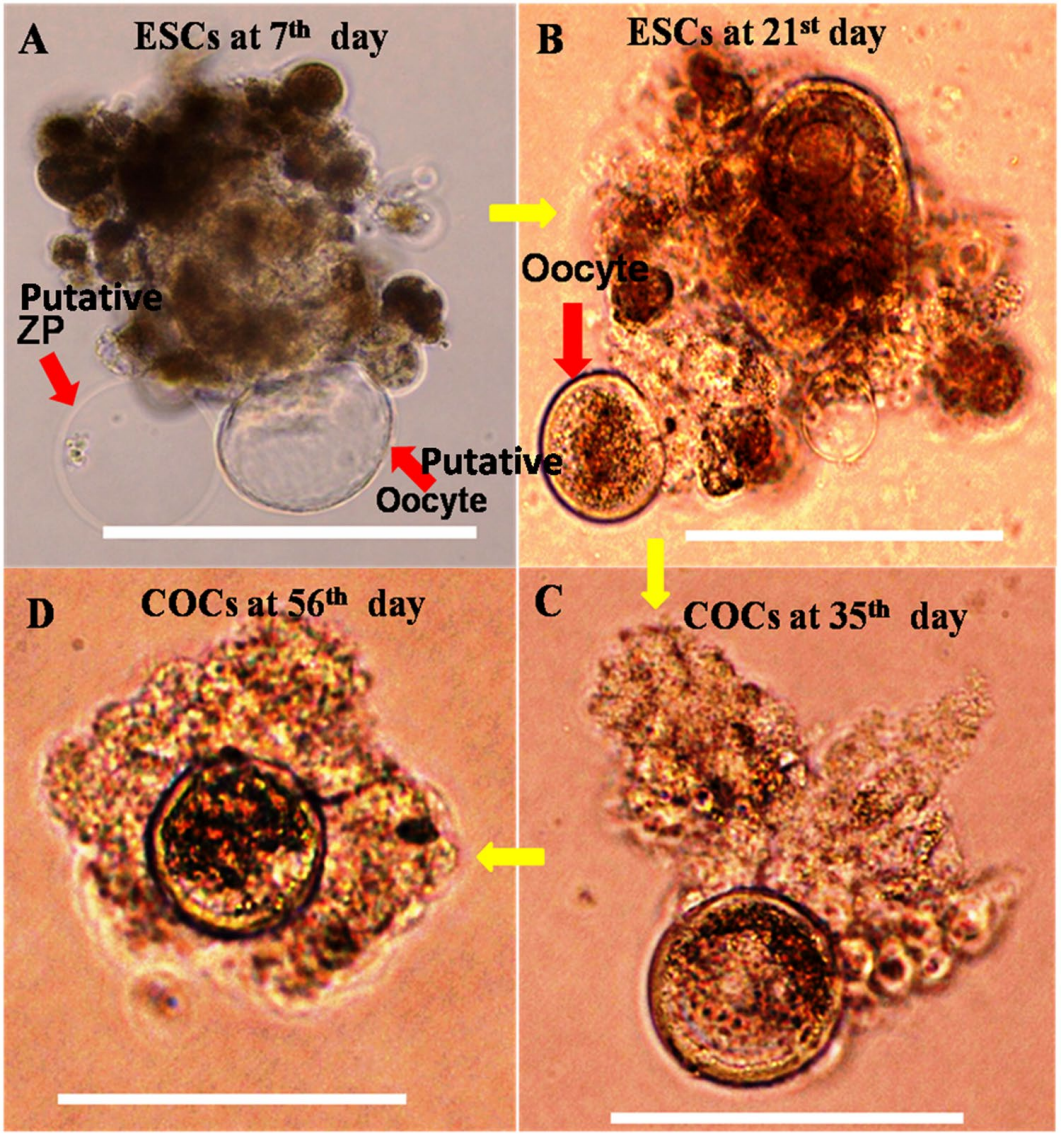
Derivation of COCs like cells directly from goat putative ESCs. **(A)** ESCs at 7^th^ day of differentiation showed putative zonapellucida (ZP). **(B)** ESCs at 21^st^ day. **(C)** COCs at 35^th^ day. **(D)** Full grown COCs at 56^th^ day of differentiation. Scale bar 100 µm.

Embryoid bodies (EBs) derived from goat ESCs have been grown for 2 months in conditioned medium supplemented with RA (1 μM) and BMP-4 (100 ng/ml). The schematic flow chart for culture and differentiation of germ cells protocol was illustrated in Fig. 1. Following *in vitro* induction, the size of the EBs were increased gradually. On the periphery of EBs, a small oocyte-like cells surrounded by 1–2 layers of cumulus cells was observed after day 21 of directed differentiation. The size of these oocytes increased gradually and accumulated layers of cumulus cells were visible after day 35 of induction. Then, the full-grown oocyte surrounded by several layers of cumulus cells was released from EB and became viable for further use (Figs. 2 and 3).

**Figure 4:**
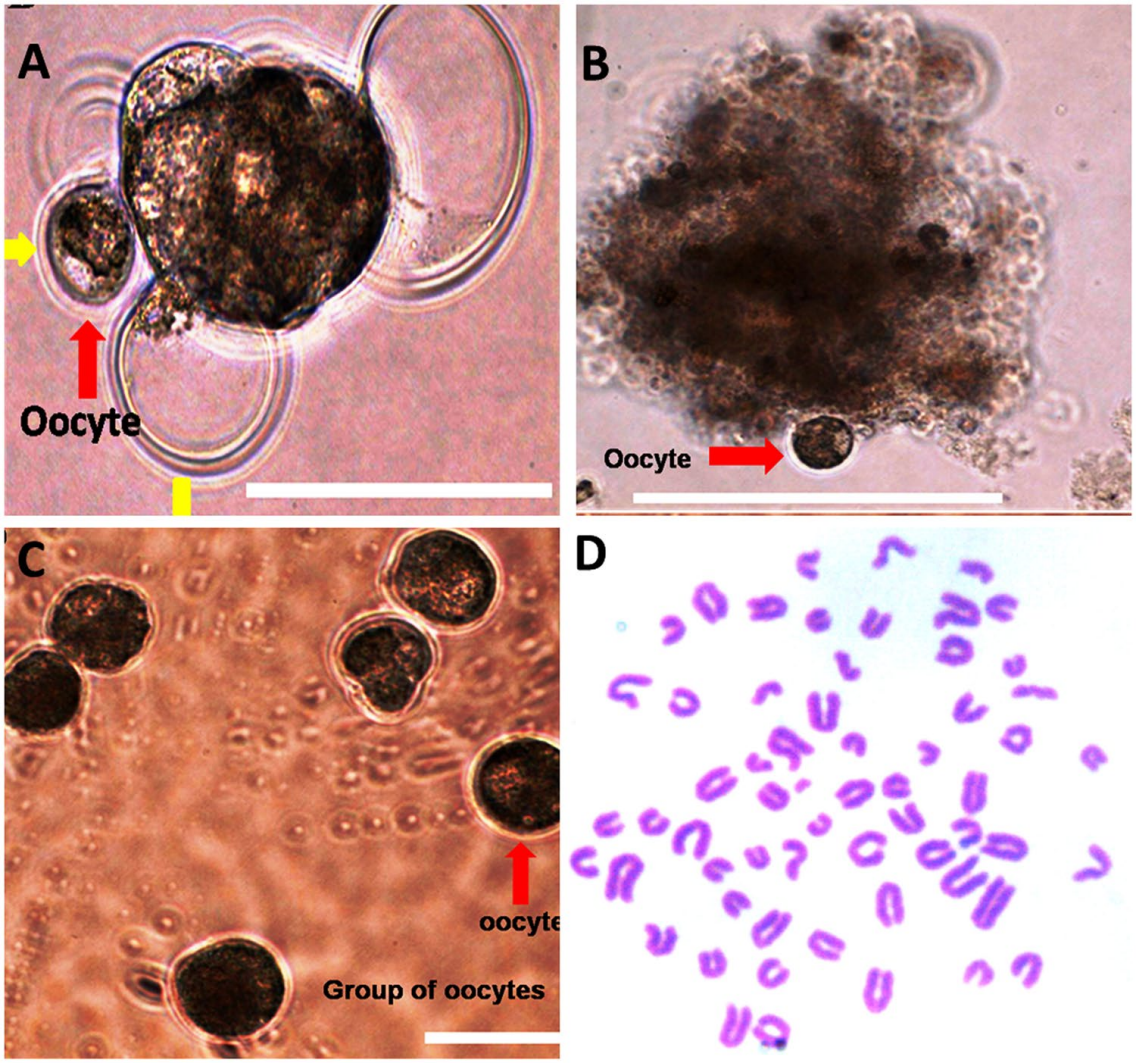
Derivation of oocyte like cells directly from goat putative ESCs and EB. **(A)** Oocyte like cells from EB. **(B)** Oocyte like cells from putative ESCs. **(C)** Group of oocyte-like cells. **(D)** Karyotyping of putative oocytes showed 30 pairs stable and normal chromosome without having any chromosomal aberration. Scale bar 100 µm.

In the present study, we have differentiated putative ESCs and ESCs-derived EBs into oocyte like cells separately (Fig. 4). The developmental potential of oocytes like cells into COCs is higher and quicker in EBs mediated differentiation than the stem cell colonies. We have observed that following *in vitro* induction, the EBs get differentiated into oocyte first, and then recruited adjacent somatic cells from the differentiating culture to form COCs. We were able to differentiate ESCs into a large number of oocytes *in vitro* (Fig. 4C) with different degree of potential (Table 1).The EBs mediated differentiation was robust, repetitive and quicker than the other. More number of COCs like cells was generated from EBs mediated differentiation than ESCs colonies. A single EB having 1 × 10^3^ cells under induction produced 3–4 oocytes like cells, whereas the ESC colony with the same amount of cell numbers gave rise to only 1–2 oocytes like cells. In addition to that, this ESCs-derived oocyte like cells under karyotyping showed thirty pairs of stable and normal chromosome without having any significant chromosomal aberration (Fig. 4D).

**Table 1:** Comparative analysis of different culture conditions for efficient differentiation of goat putative ESCs into COCs and oocyte like cells.

Culture conditions	Total no. of EB/ESCs	No. of EB/ESCs that differentiated into putative oocyte/ COCs	No. of COCs developed into putative blastocyst
EB growing in LIF free DMEM with 20% FBS	46	0	0
EB growing in LIF free DMEM with 20% FBS, 1 µM retinoic acid and 100 ng/ml BMP-4	76	58–61 (76.13–80.02%)	7 (12.06%)
ESCs growing in LIF free DMEM with 20% FBS	65	0	0
ESCs growing in LIF free DMEM with 20% FBS, 1 µM retinoic acid and 100 ng/ml BMP-4	52	13–18 (25–34.61%)	1 (7.69%)

**Figure 5:**
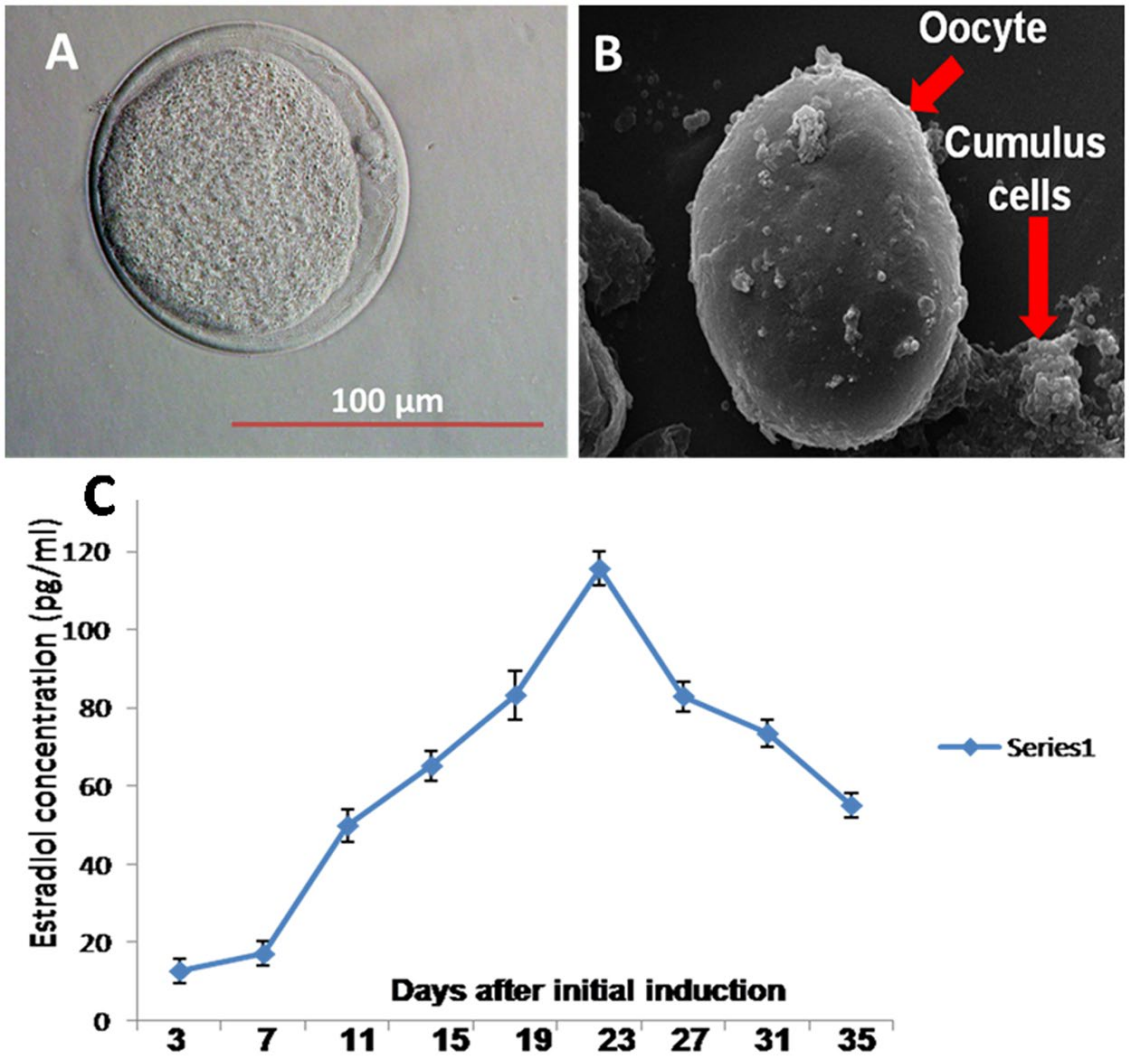
Characterization of oocyte like cells. **(A)** Oocyte-like cells exposing a distinct polar body. **(B)** Scanning electron microscopy of ESCs-derived COCs like cells revealed evenly granular cytoplasm surrounded by the different layer of cumulus cells. **(C)** 17β-estradiol assay in the differentiated culture at different days after initial induction. The peak level of 17β-estradiol (100–120 pg/ml) was observed after 19–23 days of differentiation. Scale bar 100 μm.

The generated oocyte like cells possesses distinct polar body (Fig. 5A). Moreover the scanning electron microscopy image revealed that these oocyte-like cells were surrounded by more than 2–3 layers of cumulus cells with evenly distributed granular cytoplasm (Fig. 5B). Here we employed the spent medium of differentiation at different days (3–35) of induction for 17β-estradiol assay. The presence of 17β-estradiol hormone at a minimal level (15–20 pg/ml) was detected after day 3 of induction. Thereafter, the concentration of 17β-estradiol hormone increased exponentially and reached at peak level (115–120 pg/ml) after day 19–23 of differentiation. Subsequently, the concentration of 17β-estradiol decreased gradually and reached at a minimal level (40–50 pg/ml) after day 35 of differentiation (Fig. 5C). The presence of 17β-estradiol in spent medium confirmed the ability of stem cell generated oocytes to the secret female reproductive hormone.

### Detection of germ cell specific markers in putative ESCs-derived germ cells

**Figure 6:**
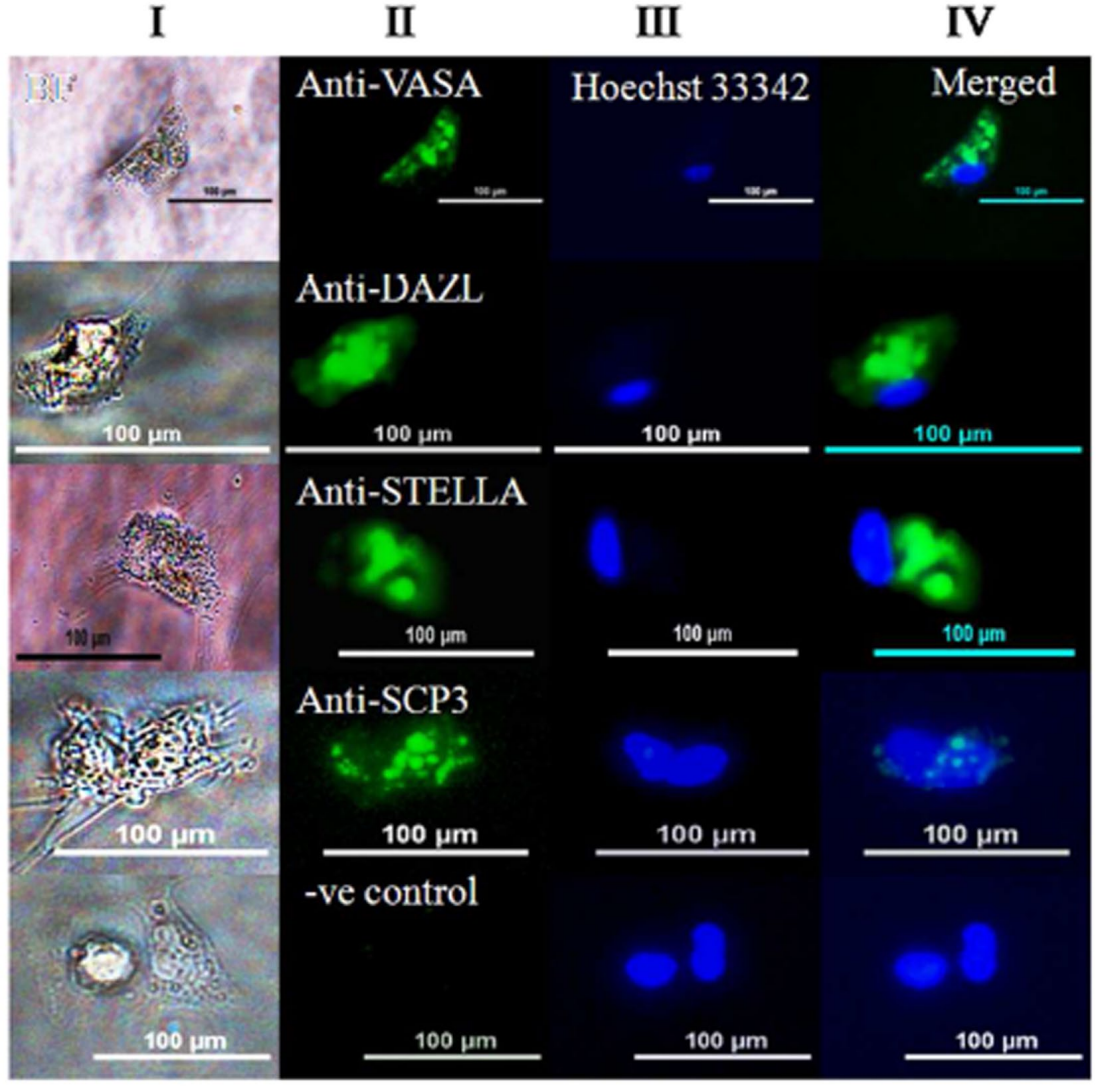
Detection of germ cell-specific markers in single PGC. (Column I): Bright field image of PGC. (Column II): Immunolocalization of germ cell-specific protein in respective PGC. (Column III): Nuclear chromatin staining of PGC with Hoechst 33342 dye. (Column IV): Merged image of column II and column III. Primary antibody was absent in negative control. Scale bar 100 μM.

The goat ESCs-derived PGCs were subjected to immunostaining with germ cell-specific markers. We have observed that some PGCs were detached from differentiated ESCs and proliferated further as a single cell. The expression of VASA, STELLA, and DAZL protein was detected in these cells, thus indicating that goat ESCs have the ability to *in vitro* differentiate to germ cells (Fig. 6). With regard to meiotic germ cell markers, SCP3 expression was detected in the ESCs-derived PGCs (Fig. 6), and since it is a definitive marker for meiosis and its presence in ESCs indicated that the differentiated cells have already reached meiosis stage of cell cycle.

### Prolificacy and chromosomal fidelity of putative ESCs-derived PGCs and oocyte-like cells

**Figure 7:**
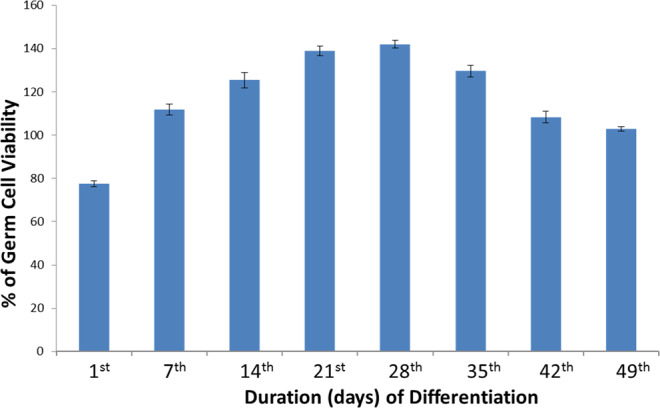
Cell viability assay of ESCs-derived germ cell with different interval of differentiation.

The ESCs-derived PGCs and oocyte like cells under karyotyping showed thirty pairs of stable and normal chromosome without having any significant chromosomal aberration (Fig. 4D). No aneuploidy and chromosomal translocation were observed in these cells. The ESCs-derived PGCs were further examined for their prolificacy by XTT [sodium 3,3′-[1(phenylamino) carbonyl]-3,4-tetrazolium]-3is (4-methoxy-6-nitro) benzene sulfonic acid hydrate] assay. The absorbance was recorded in a NanoQuant microplate reader at a wavelength of 450 nm. The ESCs-derived PGCs at the 28^th^ day of differentiation showed highest cell proliferation and possessed 141.96% cell viability (P = 0.0005) (Fig. 7). Gradually the viability of these cells decreases and at 49^th^ day of differentiation, the viability was at 102.87% (P = 0.0005).

### Characterization of putative ESCs-derived COCs, and oocyte-like cells

**Figure 8:**
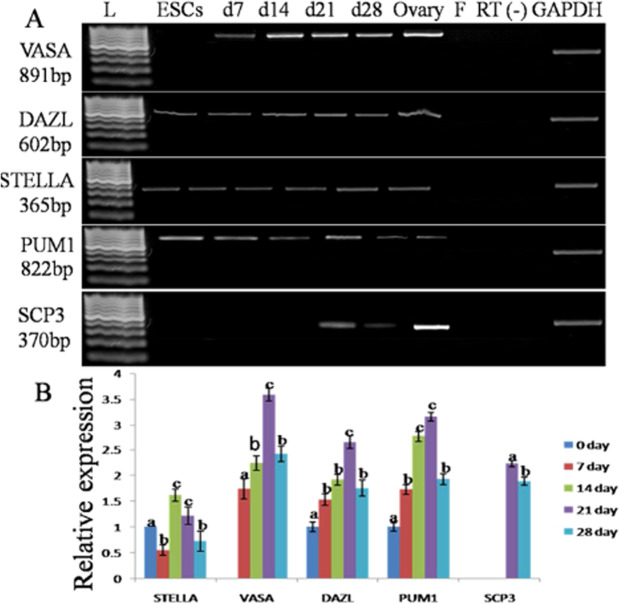
Characterization of ESCs-derived PGCs. **(A)** RT-PCR analysis of PGCs specific markers at different days (7–28) of differentiation. Expression of SCP3 was evident after 21 days of differentiation. Ovary tissue served as a positive control. Fibroblast cells and RT (−) served as negative control. GAPDH act as an internal positive standard. **(B)** Mean ± SEM expression of STELLA, VASA, DAZL, PUM and SCP3 in primordial germ cells after different days of differentiation. The *a, b and c* values having different superscripts in the same column differed significantly (P < 0.05).

**Figure 9:**
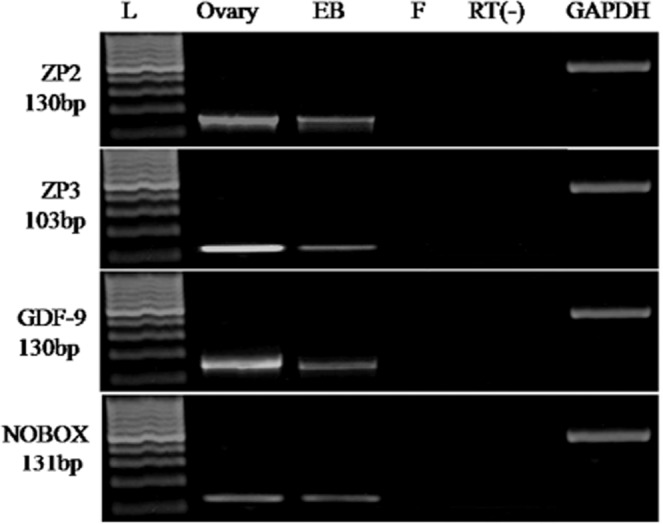
Detection of oocyte-specific markers in differentiating EBs. Ovary tissue served as a positive control. Fibroblast cells and RT (−) served as negative control. GAPDH act as internal positive standard (463 bp). Lane L: 100 bp DNA ladder.

The goat ESCs after the different interval of induction was subjected to RT-PCR analysis to detect the PGCs specific transcripts. The presence of VASA, DAZL, STELLA, PUM1, and SCP3 transcripts was observed in these cells (Fig. 8A). The transcripts of DAZL, STELLA, and PUM1 gene were detected in both undifferentiating goat ESCs and EBs at the successive interval of induction, whereas VASA and SCP3 mRNAs were absent in ESCs. A positive amplification for VASA gene was observed after day 7 of differentiation. But SCP3 transcripts were detected after day 21 (Fig. 8A). The goat ESCs after a different interval of induction were subjected to analysis the relative abundances of germ cell-specific marker by semi-quantitative RT-PCR. The transcripts of VASA, STELLA, DAZL, and PUM1 genes were detected after day 7 of differentiation (Fig. 8B). The mRNA abundance for all these markers gradually increased after day 7 of differentiation, reached the peak after day 21 and then decreased further. A positive amplification for SCP3 gene was detected after day 21 of differentiation. However, the relative mRNA abundance for VASA gene was higher than STELLA, DAZL, and PUM1 in the same duration of differentiation. The expression of zonapellucida (ZP2 and ZP3) and oocytes specific markers (GDF9, NOBOX, ZP2, and ZP3) was detected after day 21 of differentiation (Fig. 9).

### Developmental competency of putative ESCs-derived COCs like cells

**Figure 10:**
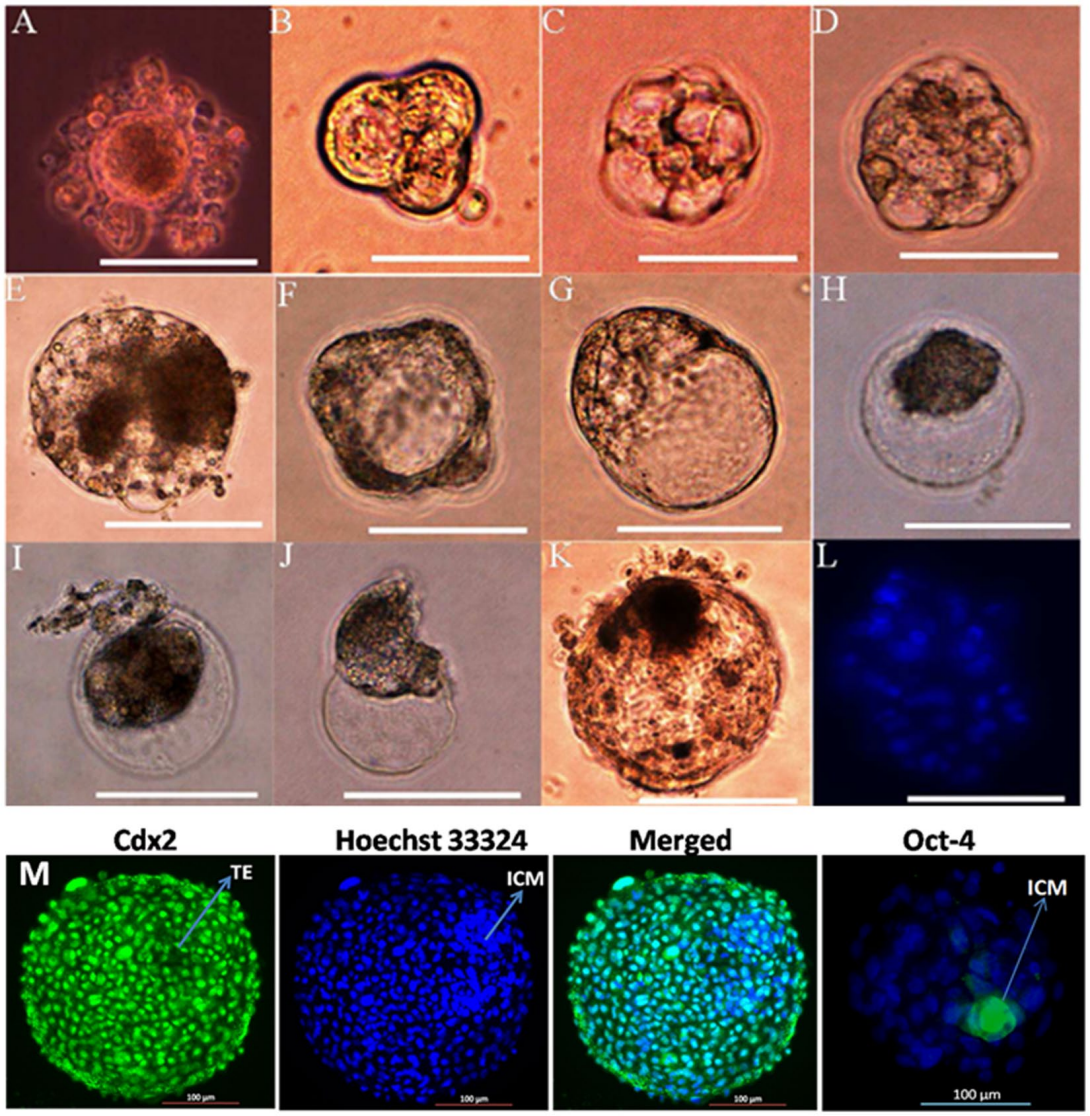
Developmental competency of ESCs-derived oocyte-like cells. **(A)** The growth of parthenogenetically activated oocytes into 2 cells stage. **(B)** 4 cells stage. **(C)** 8 cells stage. **(D)** 16–32 cells stage. **(E)** Morula stage embryo. **(F)** Early blastocyst stage embryo. **(G)** Late blastocyst cell stage embryo. **(H)** Late blastocyst containing shrunken blastomeres. **(I)** The initial stage of hatching blastocyst. **(J)** The late stage of hatching blastocyst **(K);** Hatched blastocyst containing inner cell mass. **(L)** Staining of hatched blastocyst with Hoechst 33342 dye exposed nuclear chromatins of 90–100 blastomeres. **(M)** Differential staining of blastocyst with Cdx2 and Hoechst 33342 dye exposed that Cdx2 antibody was specific for trophoectoderm cells and did not bind with inner cell mass specific cells, whereas Oct-4 antibody only binds with inner cell mass. Scale bar 100 μm.

To examine the developmental competency, we first performed *in vitro* maturation of these oocyte-like cells, and then employed parthenogenetic activation to them. After 72 h of activation, 2 cell stage embryos were appeared (Fig. 10A), but the subsequent development (4–32 cells) occurred very slowly (Fig. 10B–D). Early morula stage embryos appeared after day 6 of development and reached at compact morula stage after day 8 (Fig. 10E). Vacuolization of early blastocyst appeared after day 10 (Fig. 10F) and reached at late blastocyst stage after day 12 of development (Fig. 10G). Gradually, the blastomeres were pulled to one side of developing blastocyst and became shrunken (Fig. 10H). The zona pellucida became thinner, weaker and cracked at some portion of the blastocyst (Fig. 10I) and after day 14 of embryonic development the inner blastomeres hatched out (Fig. 10J,K). The hatched blastocyst revealed 90–100 numbers of blastomeres on staining with a DNA binding specific dye, Hoechst 33324 (Fig. 10L).

In order to evaluate the quality and efficiency of parthenogenetically produced embryo into further development, the parthenogentically produced blastocyst like cells were employed for trophoectoderm (Cdx2) and inner cell mass specific (Oct-4) staining. Cdx2 antibody is specific for trophoectoderm cells and does not bind with inner cell mass specific cells, whereas pluripotency specific markers Oct-4 specifically binds with inner cell mass as evident in Fig. 10M.

## Discussion

Cells have the potential to be differentiated into the three germ layers (ectoderm, endoderm and mesoderm) both *in vitro* and *in vivo*. Nevertheless, a few studies suggested that pluripotent stem cells exhibit the greater potential for differentiation into germ cell lineages. However, no report has been available on differentiation of embryonic stem cell into germ cells, especially into oocytes. Therefore, the goal of the current study was to evaluate the *in vitro* differentiation potential of goat embryonic stem cells into COCs.

During EB mediated ESCs differentiation, several tissue-specific transcripts and proteins are differentially expressed in a characteristic manners which mimics *in vivo* embryogenesis. Therefore, the above model system has been used earlier to productively obtain oocyte-like cells^9,14,15,20–22^. The oocytes were further fertilized to produce live offspring^23^. Within the ovary, appropriate PGC proliferation and subsequent differentiation has been dependent on spatial and temporal regulations of genes which in turn affected by various factors such as growth factors, signaling molecule and extracellular proteins. Therefore, ESC-derivation of pure population of germ cells and necessary supporting somatic cell components will be dependent on cell-culture conditions which would stimulate a gene-expression profile for optimal germ-cell differentiation. The molecular regulators in conditioned medium govern the differentiation and lineage specification. The primary source of growth factors in cell-culture medium is fetal bovine serum (FBS), human recombinant BMP-4 and RA. In a previous study^9^, the growth factors in FBS promoted germ cell differentiation from stem cells, however, in the present study, alone FBS is not capable of efficient germ cell differentiation. On the other hand, conditioned medium containing FBS, BMP-4 and RA are able to achieve this transformation.

Previous studies reported that mouse and human ESCs in co-culturing with BMP4-producing cells and supplemented with human recombinant BMP-4 increased the expression of VASA protein^24,25^. Further, the addition of RA *in vitro* was reported to stimulate the proliferation and meiotic initiation of PGCs in mice^26,27^. In the present study, following induction with conditioned medium, EBs readily expressed VASA protein. In case of mice, human and primates, among the various germ-cells markers, VASA has been a candidate gene to detect pre-meiotic germ-cell, as its expression was detected earlier in the primordial stage of germ cell development in comparison to that of PIWI family genes^16,28–32^. Since other germ cell markers such as PRDM1, PRMT5, DPPA3, IFITM3, GDF3, and c-KIT were expressed in somatic cells, it has been difficult to avoid contamination of other cells from putative germ cells by using only one of these markers. Therefore, we have used a cocktail of germ cell-specific markers (VASA, DAZL, STELLA, PUM1, Oct-4, and SCP3) to validate the characterization of germ cell derived from ESCs. In the present study, along with other germ cell markers, the expression of VASA gene is evident in differentiated embryonic stem cell colonies. The expression of VASA gene in EBs was increased after day 7 of differentiation, reached a peak at day 21 and thereafter decreased gradually, and this is in accordance with the reports of the previous study^16^. The efficient differentiation might be due to the cumulative effect of growth factors (BMP-4 and RA) and three-dimensional environments rendered by ultra-low attachment discs, and thus augmenting the plasticity of EBs to form germ cells. In addition to that, EBs cultured in ultra low attachment discs provided enough surface area for efficient germ cell differentiation.

To gain more evidence regarding germ cell abundance in culture, the differentiating EBs were further immunolocalized for DAZL, STELLA and SCP3 protein. SCP3 protein, a part of the axial-lateral element of the synaptonemal complex has been used as an excellent marker for detection of the onset of mammalian meiosis^33^. The Deleted in Azoospermia-Like (DAZL), which encodes an RNA binding protein, has been proved to regulate germ cell development in various species^34^. STELLA (Known as Dppa3), a maternal oocyte protein, that regulates the totipotency of embryo may play a role in primordial germ cell identity and reprogramming to generate the functional oocytes^35^. In the present study, the majority of germ cells were positively stained for VASA, DAZL, STELLA, and SCP3 protein, indicating that the germ cell precursors were at a developmental stage and comparable to that of post-migratory PGC or early PGC^36^.

In the present study, the expression of DAZL and PUM1 (Pumilo homolog) mRNA was increased after day 7, reached the peak at day 21 followed by a decreased expression on subsequent intervals of differentiation. The expression of STELLA transcripts was minimal up to day 7 of differentiation but in between day 7 to 21, its expression increased to some extent, thereafter decreased significantly. However, the expression of SCP3 transcripts was evident after day 21 of differentiation. Following induction with different growth factors, EBs expressed more transcript abundance for VASA than STELLA and DAZL gene in the same duration of differentiation and it may be due to the expression of the former factor is dispensable for the survival of germ cell in a long term culture. Regeneration of germ cells takes a longer time, thereby resulted in the heterogeneity of germ cell population^37,38^.

The progress of a meiotically competent oocyte in the female gonad requires the interaction of somatic cells or theca cells to aromatize androgen precursors into estrogen. Therefore, the detection of 17β-estradiol in spent medium provides the evidence for the functional activity of the differentiated somatic cells. We consistently found 17β-estradiol in the culture medium after day-7 which reached peaked around day-21 of induction and subsequently decreased and this trend is in accordance with the reports of the previous study^9^. Since the conditioned medium secreted female sex steroid, it is possible that growth factors secreted by the differentiated cells may be responsible for the transformation of germ cells into gametes.

This study was exclusively conducted on −XY ESCs. The derivation of goat –XX embryonic stem cell line depends upon the genotype of sperm during *in vitro* fertilization. Therefore, sexed semen is necessary to get the embryonic stem cell lines with desirable genotype. In the present study, the ESCs generated by previous investigator^39^ in our laboratory were employed for *in vitro* differentiation into a female genotype. The derivation of oocytes and blastocyst like structure does not depend upon the genotype of embryonic stem cells because it could be accomplished from both male and female embryonic stem cells^9^. The actual mechanism by which the PGC developed into either spermatogonia or oogonia is not well understood and sexual dimorphism is an outcome of both endogenous gene expression and local cellular interaction^40–42^. Although very few genes regulate gonadal establishment and sex differentiation, the SRY gene plays a significant role in male sex establishment^43^. We repeatedly check the expression of SRY gene during the course of induction but did not find it. Earlier researchers^9^ were able to detect the expression of the SRY gene in late cultures of mouse embryonic stem cells, but it was inappropriate. Because of an absence of expression of SRY gene, and which may be due to inappropriate influence of differentiation factors such as RA that involved in Sertoli cell and Leydig cell metabolism, lead to differentiation of stem cells with a female phenotype^9,13^.

In the present study, the expression of sperm receptor glycoprotein (ZP2 & ZP3) was detected in the zona pellucida of ESCs-derived oocyte-like cells. The presence of intact zona pellucida increases the possibility of above oocyte-like cells for further use in *in vitro* fertilization and somatic nuclear transfer technology. A fragile zona pellucida with a different layer of cumulus cells was also visible by the scanning electron microscopy, ameliorating the possibility of stem cell engineered oocytes not only in elevating the infertile ailments but can also be used as an *in vitro* model to study *in vitro* gametogenesis. Moreover, we have found that the ESCs-derived oocytes had sufficient mRNA abundance for ZP2, ZP3, NOBOX, and GDF-9 genes. The GDF-9, a member of transforming growth factor β (TGFβ) superfamily is responsible for early folliculogenesis and also has a critical role in theca cells and granulosa cells growth as well as in differentiation and maturation of oocyte^44,45^. Similarly, NOBOX a homeobox gene, preferentially expressed in oocytes is essential for folliculogenesis and regulation of other oocytes-specific genes^46,47^. The presence of NOBOX and GDF-9 transcripts gives a hint that the ESCs-derived oocyte-like cells are capable enough for further development^48^.

External activating agents (Ca^2+^ionophore and DMAP) induced sufficient stimuli to accomplish a successful parthenogenetic activation of these oocyte-like cells. In the earlier study^9^, mouse ESCs were successfully differentiated into oocytes, which further parthenogenetically developed into healthy blastocyst stage embryos. In another study, ESCs and induced pluripotent stem cells were differentiated into primordial germ cell-like cells^23^. Following aggregation with female gonadal somatic cells, these primordial germ cells undergo X-reactivation, imprint erasure, cyst formation, and exhibit meiotic potential. Upon transplanted under mouse ovarian bursa, the primordial germ cell-like cells in reconstitute ovary developed into mature oocytes which on *in vitro* fertilization, gave birth to live fertile offspring. Here, we are parthenogenetically able to develop the ESCs-derived oocyte-like cells into a blastocyst and hatched blastocyst.

## Materials and Methods

### Animal ethics

All experiments were conducted as per the guidelines and regulations of the Institute Animal Ethics Committee, ICAR-National Dairy Research Institute (NDRI), Karnal, India. This study was approved by the Institute Animal Ethics Committee, NDRI, India and all methods were performed in accordance with the relevant guidelines and regulations. In this study, we cultured the embryonic stem cells from *in vitro* fertilized goat blastocysts and directed differentiated into putative oocytes with the protocol. These *in vitro* produced putative oocytes were further parthenogenetically activated to produce hatched blastocysts.

### Chemicals

Chemicals and reagents used for this study were purchased from Sigma-Aldrich Chemical Company (Spruce Street, St. Louis, MO, USA). The reagents purchased from other than Sigma-Aldrich Chemical Company were mentioned in the text. The primary antibodies such as VASA (Cat. no. sc-48705), DAZL (Cat. no. PA5–18404), STELLA (Cat. no. sc-67250), SCP3 (Cat. no. sc-33195), ZP2 (Cat. no. sc-23714), ZP3 (Cat. no. sc-23715), Cdx2 (Cat. no. sc-166830) and FITC labelled IgG secondary antibodies (Cat. no. sc-2342) were procured from Santa Cruz Biotechnology Company (Finnell Street, Dallas, Texas, USA). Moreover, the primary antibody DAZL (Cat. no. PA5–18404) was procured from Pierce Biotechnology Company (Rockford, USA).

### The culture of putative ESCs

The ESCs generated by previous investigator^39^ in our laboratory were used for this study. These colonies were co-cultured in feeder layer made up of goat fetal fibroblast. Embryonic stem cell culture medium which comprises of Knock out DMEM, 15% knock out serum replacement, 0.5 mg/l L- glutamine, 1% (100×) non-essential amino acid, 100 ng/ml bovine fibroblast growth factor, 1000 IU/ml leukemia inhibitory factor and 1% V/V gentamycin was used for ESCs proliferation at 37.5 °C temp 5% CO_2_ in air.

### Genotyping of putative ESCs

The sexing or genotyping of ESCs colonies was performed as described by earlier reports^49^. The ESCs colonies were manually sliced into very small pieces and cultured in a hanging drop (25 µl) of EB specific culture medium (DMEM supplemented with 5% knock out serum replacement, 0.5% (100×) non-essential amino acid and 1% essential amino acid) at 37 °C in 5% air for 3 days to develop into embryoid bodies.

### Differentiation of putative ESCs into PGCs and COCs like cells

Two ways approach was employed for *in vitro* differentiation of ESCs colonies. First, culture of stem cell colonies in feeder-free system and second, a three-dimensional culture of EBs in ultra-low attachment 6 well plates (Corning, USA) at 37 °C in 5% air. Freshly prepared conditioned medium (DMEM supplemented with 20% FBS, 1 µM retinoic acid, 100 ng/ml BMP-4, 0.5% (100×) non-essential amino acid and 1% essential amino acid) was added every 3 days interval for four weeks. The differentiated cells were further examined to detect the expression the PGC specific markers (VASA, STELLA, DAZL, and SCP3) by Immunostaining and RT-PCR (Table 2). In addition to that, after 4 weeks of induction, the differentiated ESCs were examined to detect the expression of oocyte-specific markers such as GDF-9, NOBOX, ZP2, and ZP3 (Table 2).

**Table 2:** List of primers used in the study.

Gene name	Forward primer (5′-3′)	Reverse primer (5′-3′)	Melt. temp.	Prod. length (bp)	Accession number
**X- and Y- chromosome specific markers**					
*AMELOGE-NIN*	CAGCCAAACCTCCCTCTGC	CCCGCTTGGTCTTGTCTGTTGC	58	AMELX-262 AMEL-Y-202	
*SRY*	CGAAGACGAAAGKTGGCTCT	TGTGCCTCCTCAAAGAATGG	58	SRY-Y-162 SRY-X-0	
**Primordial germ cell specific markers**					
*VASA*	CTGGTGGCATTTTTGGTTCT	GCTGTTCCTTTGATGGCATT	58	891	NM_001007819
*STELLA*	AACCCAACCTGGACCCTAGA	TGGAATCTTCGCACTCTTGA	56	365	EF446905.1
*DAZL*	TCAGCTACCACCAGCCAAG	GCTCCGGTGTCAACTTCATT	56	602	NM_00108172 5.1
*PUM1*	AGAATGGGATTGACGCAGAC	AGTAAGCAGCAGGAGCCAAG	58	822	NM_001193123.1
*SCP3*	AGTGGTTCAGAGGAGGATGC	CCTTTTGTTGCTGTCGAAAC	58	370	BC102433.1
**Oocyte specific markers**					
*ZP2*	TGCCACACACATGACTCTCA	GAGGCCATTTGCTATTTCCA	59	130	HM631686.1
*ZP3*	CGAGAAGATGACACCCACCT	GCAGTGGTCCACGAACAGT	58	103	HM631711.1
*GDF9*	AGCTGAAGTGGGACAACTGG	TGGATGATGTTCTGCACCAT	56	130	HM462268.1
*NOBOX*	AGGCTCTTCCAAGATGACCA	CCATTCATTTTTCGCCACTT	58	131	XM_002687136.2

### Parthenogenetic activation of goat putative ESCs-derived COCs like cells

The ESCs-derived COCs like cells were cultured for 27 h in maturation conditioned medium at 37 °C under 5% CO_2_ in air which comprises of TCM-199, 10% estrus goat serum, 10% goat follicular fluid, 10 µg/ml luteinizing hormone, 5 µg/ml follicle stimulating hormone, 5 µg/ml 17b-estradiol, 3 mg/ml bovine serum albumin and 1% V/V Gentamicin. 27 h post maturation putative oocytes were further activated parthenogenetically as reported earlier^50^ with some minor modifications. Briefly these oocytes treated by 2 µM Ca^2+^ionophore (Ionophore A23187) for 7 min in embryo development medium (EDM). EDM comprises of TCM-199, 2.0 mM L-glutamine, 0.2 mM sodium pyruvate, 50 μg/ml gentamicin. The processed putative oocytes were again incubated in EDM supplemented with 2 mM 6-dimethylamino purine for 4 h after thorough washing. There after these oocytes were incubated in research vitro cleavage medium (RVCL, Cook, Australia) for further embryonic development.

### Cell proliferation assay

The proliferation and viability of ESCs derived PGCs cells were assayed by using XTT cell proliferation assay kit (Catalog No. 10010200, Cayman Chemical Company, MI, USA) as per manufacturer’s instruction with some minor modifications. Briefly freshly prepared DMEM was added to the six-well culture dish having ESCs derived PGCs after discarding the spent medium from these differentiated cultures and allow to pellet by centrifugation at 1000 rpm for 5 min. Again fresh DMEM was added to the pellet to make a suspension of differentiated cells and 100 μl of it was placed in a 96-well culture plate at a density of 0.5 × 10^5^ cells/well for 24 h at 37 °C with 5% CO_2_ in air. To each well freshly reconstituted XTT mixture (10 μl) was added, mixed well and incubated at 37 °C with 5% CO_2_ in air for 2 h. NanoQuant microplate reader was used to measure the absorbance of sample at a wavelength of 450 nm and the data from three repeated experiments were statistically analyzed by t-test. A p value (<0.05) was considered statistically significant.

### Evaluation of chromosomal stability of goat putative ESCs-derived COCs like cells

The ESCS derived COCs like cells without having polar body was employed for karyotyping according to the procedure of earlier reports^51^ with some minor modifications. Putative oocytes were incubated in 0.1 g ml^−1^ colcemid in DMEM, containing 3% FBS for 16–18 h followed by treatment with 0.5% sodium citrate for another 15–20 min. Thereafter 2 µl of above solution containing oocytes were transferred on to a clean glass slide. About 3 µl of previously chilled fixative (methanol: acetic acid, 3:1) solution was dropped from 2 ft height on to the oocytes. Subsequently the treated oocytes were stained in Giemsa (1%) solution followed by examination under a compound microscope (Nikon, Microphot-FXA, Japan).

### Scanning electron microscopy of goat putative ESCs-derived COCs like cells

For scanning electron microscopy goat putative ESCs-derived COCs like cells were fixed by immersion in a solution of 4% glutaraldehyde on 0.1 M phosphate buffer at pH 7.4 for 4 h. After an additional fixation with 1% osmium tetroxide in Sorensen’s buffer for 1 h, the cells were dehydrated by gradually increasing the ethanol concentration. Subsequently these cells were immersed in hexamethyldisilazine (HDMS) solution for 1 h and allowed to evaporate at room temperature. Thereafter the processed cells were mounted on SEM tubes followed by examination under Scanning Electron Microscope (Model name- Carl Zeiss Ag- EVO40).

### Estradiol measurements

The 17β-estradiol concentration in spent medium was assayed using estradiol EIA kit as described by manufacturer (Cayman Chemical Company, Ann Arbor, MI 48108).

### Immunocytochemistry

The adherent cells were fixed in 4% phosphate buffered paraformaldehyde solution at ambient temperature for 30 minutes. The fixed cells were permeabilized in 0.5% (V/V) Triton X-100 in Dulbecco’s phosphate buffer saline (DPBS) for another 30 min followed by three repeated washing. Afterwards it was blocked by adding 4% normal goat serum for 1 h at room temperature. Thereafter primary antibodies at a dilution of 1:100 were added and kept overnight at 4 °C. For negative control primary antibodies were excluded. After overnight incubation the cells were employed for repeated three washings in DPBS followed by adding of FITC-labelled goat anti-mouse IgG (1:1000) for 2 h at room temperature. The cells were then employed for Hoechst 33342 dye (1 µg/ml) staining for 2 min at room temperature for chromatin staining and examined under a fluorescence microscope (Diaphot, Nikon, Tokyo, Japan).

### Reverse transcriptase PCR and quantitative PCR

The putative ESCs were inspected to reveal the germ cell specific markers by reverse transcriptase polymerase chain reaction (RT-PCR). Entire RNA was isolated by using Trizol reagent (Invitrogen Corporation, Carlsbad, CA, USA) according to the manufacture’s instruction and evaluated by calculating the absorbance at 260 nm. First strand complementary DNA (cDNA) was synthesized from 500 ng of total RNA by using Revert Aid^TM^ First Strand c-DNA synthesis kit (Fermentas Life Sciences, EU). The primers used in this study were listed in Table 2. The Real-time PCR was performed to evaluate the relative abundance of PGCs development markers in a Light Cycler® 480 with software version 1.5 (Roche Diagnostics, Mannheim, Germany). All quantitative PCR runs were carried out in triplicate and individual reaction mixture was formulated in a total volume of 10 µl. The reaction mixture comprised of 2 µl of cDNA as template, 5 µl of 2× Maxima SYBR Green/ROX qPCR Master Mix (Thermo Scientific) incorporating 0.5 µM of gene-specific primer. The following PCR reaction parameters were used: 40 cycles of denaturation at 95 °C for 15 sec, annealing at 60 °C for 30 sec and extension at 72 °C for 30 sec preceded by an initial 10-min step at 95 °C to activate the polymerase. As a controlled transcript we used Glyceraldehyde-3-phosphate dehydrogenase (GAPDH) for normalizing the gene expression. Relative mRNA expression was calculated using the 2^(-ΔΔCt)^ method^52^. Fold change in expression over the control was calculated to evaluate the abundance of transcript level.

### Statistical analysis

Statistical analysis was performed using SYSTAT 12 (Systat Software, Inc., Chicago, IL, USA). Comparison of data regarding gene expression among different groups was carried out by one-way ANOVA. Data were presented as mean ± SEM. Differences were considered significant if the P value was less than 0.05. All the experiments were replicated at least four times.

### Ethical approval and informed consent

Ethical approval was taken from Institute ethics committee, ICAR-National Dairy Research Institute (NDRI), Karnal, India during the study and all methods were performed in accordance with the relevant guidelines and regulations.

## Conclusion

In conclusion, we have developed a highly robust and authenticated approach to differentiate goat embryonic stem cells into oocyte-like cells. Following aggregation with adjacent somatic cells in differentiating culture, these cells were transformed into COCs like cells. These cells were further developed into blastocysts and hatched blastocyst stage embryos parthenogenetically, therefore demonstrating that the cells are actually totipotent even *in vitro*. The present method of oocyte derivation can be employed in other species also. The present findings can be helpful to conserve the germplasm of elite animals. However, further efforts regarding the efficient derivation of oocytes with a minimum duration, designing a better differentiation protocol, and elucidation of intrinsic mechanisms for successful implantation are quite essential for successful live birth.

## Supplementary information

Supplementary information.

## References

[1] McLaren A (1984). Meiosis and differentiation of mouse germ cells. Symposia of the Society for Experimental Biology.

[2] Ginsburg M, Snow MHL, McLaren A (1990). Primordial germ cells in the mouse embryo during gastrulation. Development.

[3] Clark JM, Eddy EM (1975). Fine structural observations on the origin and associations of primordial germ cells of the mouse. Developmental Biology.

[4] Saiti D, Lacham-Kaplan O (2007). Mouse germ cells development *in vivo* and *in vitro*. Biomark Insights.

[5] Evans MJ, Kaufman MH (1981). Establishment in culture of pluripotential cells from mouse embryos. Nature.

[6] Martin GR (1981). Isolation of a pluripotent cell line from early mouse embryos cultured in medium conditioned by terato carcinoma stem cells. Proceedings of the National Academy of Sciences of the United States of America.

[7] Thomson JA (1998). Embryonic stem cell lines derived from human blastocysts. Science.

[8] Draper JS, Moore HD, Ruban LN, Gokhale PJ, Andrews PW (2004). Culture and characterization of human embryonic stem cells. Stem Cells and Development.

[9] Hubner K (2003). Derivation of oocytes from mouse embryonic stem cells. Science.

[10] Nayernia K (2006). *In vitro*-differentiated embryonic stem cells give rise to male gametes that can generate offspring mice. Developmental cell.

[11] Novak I (2006). Mouse embryonic stem cells form follicle-like ovarian structures but do not progress through meiosis. Stem Cells.

[12] Toyooka Y, Tsunekawa N, Akasu R, Noce T (2003). Embryonic stem cells can form germ cells *in vitro*. Proceedings of the National Academy of Sciences of the United States of America.

[13] Geijsen N (2004). Derivation of embryonic germ cells and male gametes from embryonic stem cells. Nature.

[14] Lacham-Kaplan O, Chy H, Trounson A (2006). Testicular cell conditioned medium supports differentiation of embryonic stem cells into ovarian structures containing oocytes. Stem Cells.

[15] Qing T (2007). Induction of oocyte-like cells from mouse embryonic stem cells by co-culture with ovarian granulosa cells. Differentiation.

[16] Yamauchi K, Hasegawa K, Chuma S, Nakatsuji N, Suemori H (2009). In Vitro Germ Cell Differentiation from Cynomolgus Monkey Embryonic Stem Cells. PloS One.

[17] Imamura M (2010). Induction of primordial germ cells from mouse induced pluripotent stem cells derived from adult hepatocytes. Molecular Reproduction and Development.

[18] Clark AT (2004). Spontaneous differentiation of germ cells from human embryonic stem cells *in vitro*. Human Molecular Genetics.

[19] Zwaka TP, Thomson JA (2005). A germ cell origin of embryonic stem cells?. Development.

[20] Nicholas CR, Haston KM, Grewall AK, Longacre TA, ReijoPera RA (2009). Transplantation directs oocyte maturation from embryonic stem cells and provides a therapeutic strategy for female infertility. Human Molecular Genetics.

[21] Kerkis A (2007). In Vitro Differentiation of Male Mouse Embryonic Stem Cells into Both Presumptive Sperm Cells and Oocytes. Cloning and Stem Cells.

[22] Rohwedel J, Guan K, Wobus AM (1999). Induction of cellular differentiation by retinoic acid *in vitro*. Cells tissues organs.

[23] Hayashi K (2012). Offspring from Oocytes Derived from *in vitro* Primordial Germ Cell–like Cells in Mice. Science.

[24] Ying Y, Qi X, Zhao GQ (2001). Induction of primordial germ cells from murine epiblasts by synergistic action of BMP4 and BMP8B signaling pathways. Proceedings of the National Academy of Sciences of the United States of America.

[25] Kee K, Gonsalves JM, Clark AT, Pera RA (2006). Bone morphogenetic proteins induce germ cell differentiation from human embryonic stem cells. Stem Cells and Development.

[26] Koshimizu U, Watanabe M, Nakatsuji N (1995). Retinoic acid is a potent growth activator of mouse primordial germ cells *in vitro*. Developmental Biology.

[27] Bowles J (2006). Retinoid signaling determines germ cell fate in mice. Science.

[28] Castrillon DH, Quade BJ, Wang TY, Quigley C, Crum CP (2000). The human VASA gene is specifically expressed in the germ cell lineage. Proceedings of the National Academy of Sciences of the United States of America.

[29] Kuramochi-Miyagawa S (2001). Two mouse piwi-related genes: miwi and mili. Mechanism of Development.

[30] Noce T, Okamoto-Ito S, Tsunekawa N (2001). Vasa homolog genes in mammalian germ cell development. Cell Structure and Function.

[31] Qiao D, Zeeman AM, Deng W, Looijenga LH, Lin H (2002). Molecular characterization of hiwi, a human member of the piwi gene family whose overexpression is correlated to seminomas. Oncogene.

[32] Lee JH, Schutte D, Wulf G, Fuzesi L, Radzun HJ (2005). Stem-cell protein Piwil2 is widely expressed in tumors and inhibits apoptosis through activation of tat3/Bcl-XL pathway. Human Molecular Genetics.

[33] Yuan L (2000). The murine SCP3 gene is required for synaptonemal complex assembly, chromosome synapsis, and male fertility. Molecular Cell.

[34] Xu EY, Moore FL, Pera RA (2001). A gene family required for human germ cell development evolved from an ancient meiotic gene conserved in metazoans. Proceedings of the National Academy of Sciences of the United States of America.

[35] Clark AT, Pera RA (2006). Modeling human germ cell development with embryonic stem cells. Regenerative Medicine.

[36] Lacham-Kaplan O (2004). *In vivo* and *in vitro* differentiation of male germ cells in the mouse. Reproduction.

[37] Van der Wee KS, Johnson EW, Dirami G, Dym TM, Hofmann MC (2001). Immunomagnetic isolation and long term culture of mouse type Aspermatogonia. Journal of Andrology.

[38] Creemers LB, den Ouden K, van Pelt AMM, de Rooij DG (2002). Maintenance of adult mouse type-A spermatogonia *in vitro*: influence of serum and growth factors and comparison with prepubertal spermatogonial cell culture. Reproduction.

[39] Garg S (2012). Cardiomyocytes rhythmically beating generated from goat embryonic stem cell. Theriogenology.

[40] Merchant-Larios H, Moreno-Mendoza N (2001). Onset of sex differentiation: dialogue between genes and cells. Archives of Medical Research.

[41] Demeestere I (2004). Effect of insulin like growth factor-I during preantral follicular culture on steroidogenesis, *in vitro* oocyte maturation, and embryo development in mice. Biology of Reproduction.

[42] Mittwoch U (2004). The elusive action of sex-determining genes: mitochondria to the rescue?. Journal of Theoretical Biology.

[43] Koopman P, Munsterberg A, Capel B, Vivian N, Lovell-Badge R (1990). Expression of a candidate sex determining gene during mouse testis differentiation. Nature.

[44] Hreinsson JG (2002). Growth differentiation factor-9 promotes the growth, development, and survival of human ovarian follicles in organ culture. Journal of Clinical Endocrinology and Metabolism.

[45] Su Y (2004). Synergistic roles of BMP15 and GDF9 in the development and function of the oocyte-cumulus cell complex in mice: genetic evidence for an oocyte-granulosa cell regulatory loop. Developmental Biology.

[46] Suzumori N, Yan C, Matzuk MM, Rajkovic A (2002). Nobox is a homeobox-encoding gene preferentially expressed in primordial and growing oocytes. Mechanism of Development.

[47] Huntriss J, Hinkins M, Picton HM (2006). cDNA cloning and expression of the human NOBOX gene in oocytes and ovarian follicles. Molecular Human Reproduction.

[48] Zhou Q (2016). Complete Meiosis from Embryonic Stem Cell-Derived Germ Cells In Vitro. Cell stem cell.

[49] Malik HN (2013). Single Blastomere Sexing of Caprine Embryos by Simultaneous Amplification of Sex Chromosome Specific Sequence of *SRY* and Amelogenin Genes. Livestock science.

[50] Jena MK (2012). Handmade cloned and parthenogenetic goat embryos -A comparison of different culture media and donor cells. Small Ruminant Research.

[51] Li GP, Liu Y, White KL, Bunch TD (2005). Cytogenetic analysis of diploidy in cloned bovine embryos using an improved air-dry karyotyping method. Theriogenology.

[52] Livak KJ, Schmittgen TD (2001). Analysis of relative gene expression data using real time quantitative PCR and the 2 (-Delta Delta C (T)) method. Methods.

