# Supplementary material for: Derivation of oocyte-like cells from putative embryonic stem cells and parthenogenetically activated into blastocysts in goat
Source: Sci Rep. 2020 Jun 22;10:10086. doi: 10.1038/s41598-020-66609-2 (PMC7308273; doi:10.1038/s41598-020-66609-2)
Supplement: Supplementary file 1 — Supplementary information. [file 41598_2020_66609_MOESM1_ESM.pdf]

Derivation of oocyte-like cells from putative embryonic stem cells and parthenogenetically activated into blastocysts in goat

Hruda Nanda Malik, Dinesh Kumar Singhal, Sikander Saini and Dhruba Malakar*

Animal Biotechnology Centre, National Dairy Research Institute, Karnal-132001, India.

*Corresponding author. Tel:+91-9416741839; E-mail: dhrubamalakar@gmail.com

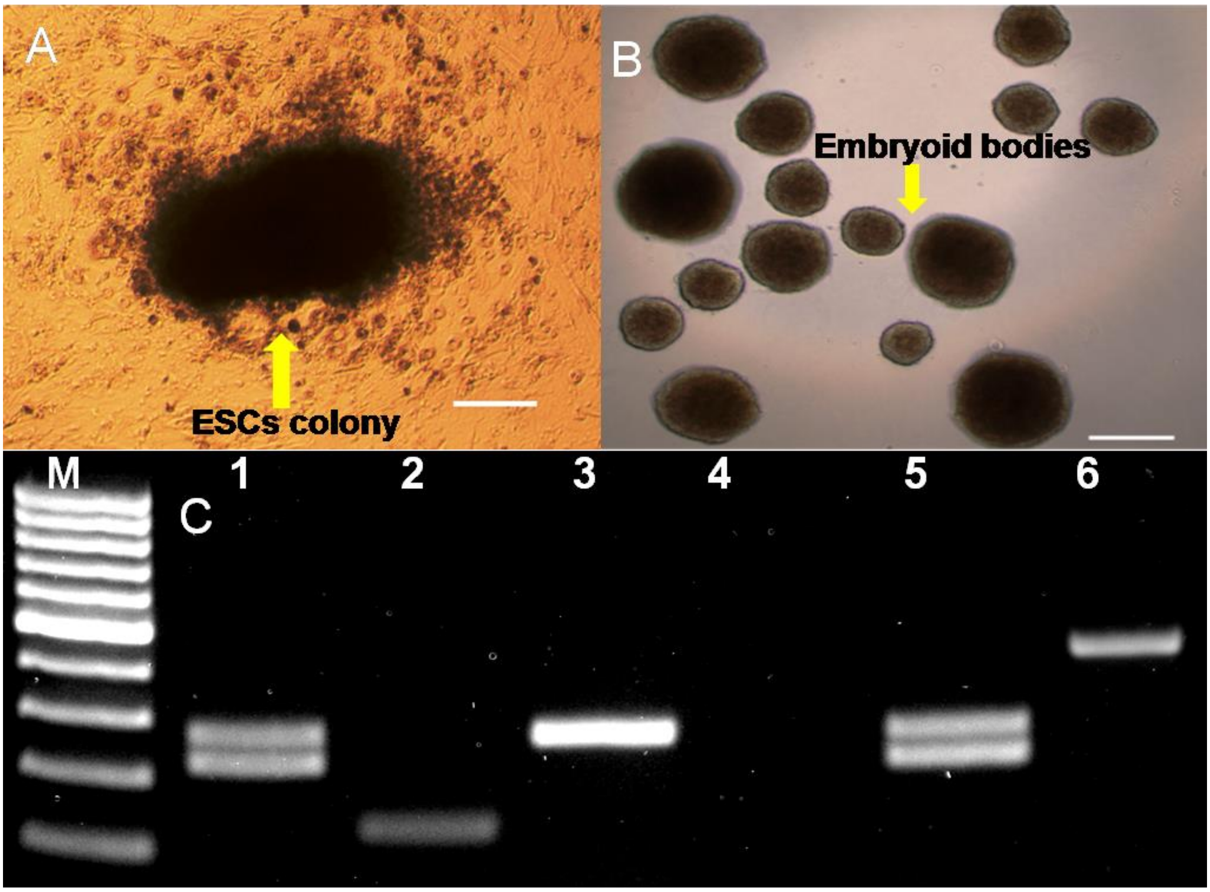

Supplementary figure 1.Establishment of goat ESCs. (A) Culture of ESCs colony on goat fetal fibroblast feeder layer. (B) Compact and dense embryoid bodies. (C) Sexing of ESCs colonies. Lane 1: Genomic DNA of ESCs colonies produced two amplified products of amelogenin gene with different sizes of 262bp (AMELX) and 202bp (AMELY) affirming the male genotype of these cells. Lane 2: Genomic DNA of ESCs colonies produced 162bp of amplified product of SRY gene (male specific). Lane 3: Female fibroblast cells; Lane 4: Negative control (No template); Lane 5: Male fibroblast cells; Lane 6: GAPDH served as an internal positive standard (463bp); Lane M: 100bp DNA ladder. Scale bar 50 μm.
